# Cognitive Dysfunction among HIV Positive and HIV Negative Patients with Psychosis in Uganda

**DOI:** 10.1371/journal.pone.0044415

**Published:** 2012-09-06

**Authors:** Noeline Nakasujja, Peter Allebeck, Hans Agren, Seggane Musisi, Elly Katabira

**Affiliations:** 1 Department of Psychiatry, College of Health Sciences, Makerere University, Kampala, Uganda; 2 Department of Public Health Sciences, Karolinska Institute, Stockholm, Sweden; 3 Department of Psychiatry and Neurochemistry, Institute of Neuroscience and Physiology, University of Gothenburg, Gothenburg, Sweden; 4 Department of Medicine, College of Health Sciences, Makerere University, Kampala, Uganda; Chiba University Center for Forensic Mental Health, Japan

## Abstract

**Background:**

Cognitive impairment is an established phenomenon in HIV infected individuals and patients that have psychosis. However there is need to establish the severity of the impairment if patients are co morbid with both conditions.

**Aim:**

To compare cognitive function among HIV positive individuals and HIV negative individuals with psychosis.

**Methods:**

We recruited patients with psychosis at two national referral hospitals. A standardized demographics questionnaire and psychiatric, physical, and laboratory assessments were conducted. Types of psychosis were diagnosed using the Mini International Neuropsychiatric Inventory-PLUS while cognitive functioning was determined using the Mini mental state examination (MMSE) and a neuropsychological test battery. Follow-up assessments on cognitive function and severity of psychiatric illness were performed at 3 and 6 months. Pairwise comparison and multivariable logistic regression analysis were used to determine the differences between the HIV positive and HIV negative individuals.

**Results:**

There were 156 HIV positive and 322 HIV negative participants. The mean age was 33 years for the HIV positive group and 29 years for the HIV negative group (p<0.001). The HIV positive individuals were almost three times (OR = 2.62 CI 95% 1.69–4.06) more likely to be cognitively impaired on the MMSE as well as the following cognitive tests:- WHO-UCLA Auditory Verbal Learning Test (OR 1.79, 95% CI 1.09–2.92), Verbal Fluency (OR 3.42, 95% CI 2.24–5.24), Color Trails 1 (OR 2.03, 95% CI 1.29–3.02) and Color Trails 2 (OR 3.50 95% 2.00–6.10) all p = 0.01. There was improvement in cognitive function at follow up; however the impairment remained higher for the HIV positive group (p<0.001).

**Conclusion:**

Cognitive impairment in psychosis was worsened by HIV infection. Care plans to minimize the effect of this impairment should be structured for the management of individuals with HIV and psychosis.

## Introduction

Cognitive dysfunction in patients having primary psychiatric illness like schizophrenia has been well documented [1], [2]. The cognitive functioning of an individual with psychosis is affected by a number of factors including the severity of psychosis and anti psychotic medication being taken [3]. In many patients the cognitive impairment is not secondary to delusions or effects of hallucinations but may rise from the lack of motivation the patients experience [4]. Though cognitive dysfunction does not occur in all patients with psychosis [5], the dysfunction is common among patients with HIV associated psychosis, [6], [7].

The prevalence of HIV dementia among HIV positive individuals has decreased from 30–40% before the introduction of highly active antiretroviral therapy to 10–15% in settings with adequate access to the medication [8], [9], [10]. In Uganda, ambulant patients attending an HIV outpatient clinic were found to have an HIV dementia prevalence rate of 31% especially if they had low CD4 count and were of older age [11]. While the severity of impairment may decrease with antiretroviral therapy (ART), the prevalence of any degree of cognitive impairment even after the use this medication remains as high as 40–70-% [12], [13].

The debilitation that occurs in HIV positive individuals who are cognitively impaired hinders their management as they may fail to adhere to their treatment regimen, the situation being further worsened if the person has a psychotic condition. In resource constrained settings, lack of clear guidelines at primary care centers and the scarcity of ART results in delayed treatment [14] creating a dilemma in the management of HIV and related conditions like psychosis, even when there is evidence that ART improves the symptoms of psychosis and cognitive impairment [12], [15]. Understanding the level of cognitive function in patients that develop psychosis would create insight into ways of managing them. This study set out to compare the cognitive deficits among psychotic HIV positive and psychotic HIV negative individuals.

## Methods

We consecutively recruited 478 patients who were admitted at Mulago and Butabika national referral hospitals in Kampala, Uganda between February 2008 and April 2009. Ethical approval for conduction of the study was received from the Uganda National Council for Science and Technology as well as the Makerere University Research and Ethics Committee.

Individuals were included in the study if they had features of psychosis, were aged 18–59 years old, were resident within a radius of 30 km of the city centre, and gave written informed consent to participate in the study. We excluded individuals who had any known medical condition other than HIV and its complications e.g. syphilis that could be related to the manic episode, a recent onset of severe headache or substance dependency. The participants received a standardized demographics questionnaire, psychiatric, physical, and laboratory assessments as described below.

### Assessments for Psychiatric Illness

The Mini International Neuropsychiatric Inventory-PLUS (MINI-PLUS) instrument was used to diagnose psychiatric illnesses: mania, depression, schizophrenia and psychosis not otherwise specified (PSY NOS) [16]. The severity of the different disorders was determined at baseline, 3 and 6 months using the Young Mania Rating Scale (YMRS) [17], the Brief Psychiatric Rating Scale (BPRS) [18] and the Patient Health Questionnaire (PHQ-9) [19]. The participants’ consent to continue in the study was again sought when the patients came for the follow up interviews.

### Assessment for Cognitive Function

The cognitive function for the HIV positive individuals was tested using the International HIV Dementia Scale (IHDS) [20]. Both HIV positive and HIV negative groups received a battery of neuropsychological tests for evaluating the different cognitive domains. The tests included: WHO UCLA Auditory Verbal Learning Test for verbal memory, learning and recall; Symbol Digit Modalities Test for visual motor coordination; Animal Recall for verbal fluency; the Digit Span backward (WAIS III) for working memory; Digit Span forwards(WAIS III) for attention; Color Trails 1 and Color Trails 2 for abstraction/executive and speed of information processing. Each test score was standardized to normal, 1 or 2 standard deviations (sd) from the mean in comparison to normative values of the general non HIV, non psychotic population [21]. The three following categories of cognitive impairment were created; normal if scores were not deviating from the mean; mild if an individual had 1.0 sd in any of the tests up to a maximum of 6 tests and severe if an individual had I.0 sd in any of the tests and in addition had 2.0 sd in one or more tests. The neuropsychological tests were repeated at 3 and 6 months.

### Laboratory Evaluations

The HIV testing was done using DETERMINE I/II (Abbot Japan Cp. Ltd, Minato-ku, Tokyo, Japan), it was validated using STAT PAK (ChemiBio Diagnostics System, Inc., Medford, USA) and UNIGOLD (Trinity Biotech Plc, Bray Co Wicklow, Ireland) test kits. HIV pre and post-test counseling was done for all patients. For individuals who were found to be HIV positive and met criteria for starting ART, this treatment was initiated at the HIV treatment clinics at Mulago or Butabika hospitals. Other laboratory evaluations included a full blood count, CD 4 count, cryptococcal antigen, toxo titers and Venereal Disease Research Laboratory (VDRL).

The study interviews were carried out in the locally spoken language Luganda or English. The patients continued to receive routine psychiatric care in the hospital even after being enrolled into the study. Three hundred seventy eight (79%) patients returned for the 3 month and 302 (63%) returned for the 6 months visit. The loss to follow up at 6 months was 36.8%.

### Data Analysis

The data was analysized using STATA version 10, (StataCorp, College Station, TX USA). Chi square test and Fishers exact test were used to determine the difference in type of psychiatric illness, MMSE scores and to evaluate differences on neuropsychological performance for the HIV positive and HIV negative groups. The level of cognitive function was determined by the presence or absence of impairment on specific cognitive domains. The likelihood of cognitive impairment was determined using logistic regression using a stepwise approach while controlling for age, gender, HIV status and educational level.

## Results

The HIV positive individuals were older, mean (SD), 33 years (8.24) than the HIV negative individuals 29 years (8.07); (p<0.001). Most individuals 372 (77.82%) had more than 7 years of education and there was no statistical difference between the HIV positive and HIV negative groups p = 0.424. The average CD4 count in the HIV positive group was 305 cell/uL. Only 7(4%) of the HIV positive individuals were at WHO clinical stage 4 i.e. AIDS. Only 43 (27.5%) HIV positive individuals were on ART at baseline and 52 (33.3%) were on it by the 6 month. All patients were taking antipsychotic medication that included chlorpromazine, haloperidol stelazine and or an antidepressant like a tricyclic antidepressant or fluoxetine depending on the disorder they were being treated for. We found that 67 (43%) of the HIV positive individuals did not have a prior episode of mental illness and pair wise comparison with the HIV negatives 88 (27%) who had no prior episodes was statistically significant, p<0.001. The mean score on the MMSE was 20.32 (5.13) for the HIV positive and 22.87(4.79) for the HIV negative group. The mean IHDS score was 5.8(2.3). The psychiatric diagnoses by gender are presented in table 1; mania was the commonest type of psychosis for both males and females. Psy NOS occurred more in the HIV positive group (p<0.001). There was no history of prior episodes of psychiatric illness in 10 (62.5%) of the HIV positive individuals who had Psy NOS. At the baseline MMSE evaluation, there were more cognitively impaired individuals within the HIV positive group 64.7%vs 35.3% than in the HIV negative group 49.4% vs 50.6%, (p<0.000). The HIV positive individuals were almost three times (OR = 2.62 CI 95% 1.69–4.06) as likely to be cognitively impaired. The females 270 (58.82%) were more impaired than the males 189 (41.18%), p = 0.018. All tested cognitive tests apart from digit span were more likely to be impaired in the HIV positive group (table 2). The odds of impairment on each of the tests are presented in table 3. Adjusted odds revealed female gender (OR 2.89, 95% 1.05–7.92), p = 0.038 and older age (OR 1.62, 95% 0.59–4.45), p = 0.34 to be associated with cognitive impairment (table 4). At the 3 months follow up, the mean MMSE score was 22.93 in the HIV positive group and 24.41 for the HIV negative group while at 6 months it was 22.15 and 24.25 respectively. The categories for the levels of cognitive impairment at follow up are summarized in table 5. There was also improvement in psychiatric symptoms in all patients p<0.001.

**Table 1:** Type of psychosis by gender.

Type of psychosis	Females N = 276			Males N = 202		
	HIV + n (%)	HIV– n (%)	p-value	HIV + n (%)	HIV– n (%)	p-value
Mania	78 (69.64)	112 (68.29)	0.812	25 (56.82)	97(61.39)	0.583
Major Dep*	14 (12.50)	26 (15.85)	0.437	7 (15.91)	8 (5.06)	0.015
Psy NOS†	6 (5.36)	0 (0 )	0.000	8 (18.18)	2 (1.27)	0.000
Schizophrenia	16 (14.29)	29 (17.68)	0.453	4 (9.09)	51 (32.28)	0.002

* Major Dep: represents Major depressive disorder,

† Psy NOS: represents Psychosis not otherwise specified.

**Table 2:** Neuropsychological test performance among HIV positive and HIV negative individuals at baseline.

	HIV+	HIV-	p value
Verbal memory VLT RAI	5.37	5.98	0.13
Verbal memory Delayed recall	5.19	5.78	0.02*
Symbol Digit	18.70	20.28	0.13
Verbal Fluency	10.06	11.31	<0.01*
Digit Span Forward	5.84	6.46	<0.01*
Digit Span Backward	3.37	3.16	0.20
Color Trails 1 (seconds)	146.50	104.84	<0.00*
Color Trails 2 (seconds)	211.09	182.47	<0.00*

VLT RAI; Verbal Learning Test recall after interference.

* statistically significant.

**Table 3:** Estimated odds ratios of being impaired in specific cognitive domains.

Domain	Variable	OR	P value	95% CI
**Verbal memory**	HIV negative	Referent		
	HIV positive	1.79	0.019	1.09–2.92
**Symbol Digit**	HIV negative	Referent		
	HIV positive	1.08	0.711	0.72–1.62
	Age <30 years	Referent		
	Age >30 years	1.61	0.012	1.10–2.36
	Educ <7 years	Referent		
	Educ >7 years	1.68	0.020	1.08–2.61
**Verbal Fluency**	HIV negative	Referent		
	HIV positive	3.42	0.000	2.24–5.24
	Male	Referent		
	Female	1.18	0.393	0.80–1.74
**Digit Span**	HIV negative	Referent		
	HIV positive	1.18	0.422	0.78–1.78
	Age <30 years	Referent		
	Age >30 years	1.01	0.959	0.68–1.48
**Colour Trails 1**	HIV negative	Referent		
	HIV positive	2.03	0.002	1.29–3.02
	Male	Referent		
	Female	1.15	0.469	0.77–1.71
**Color Trails 2**	HIV negative	Referent		
	HIV positive	3.50	0.000	2.00–6.10
	Age <30 years	Referent		
	Age >30 years	1.51	0.063	0.97–2.35
	Male	Referent		
	Female	1.19	0.408	0.78–1.83

Adjusted for all other covariates for each cognitive domain.

**Table 4:** Odds ratios for cognitive impairment in patients with psychosis.

		Non adjusted OR	P value	Adjusted OR	P value
		95% (CI)		95% (CI)	
**HIV status**	HIV negative	1		1	
	HIV positive	2.66 (0.77–9.29)	0.124	1.95 (0.053–7.01)	0.309
**Gender**	Male	1		1	
	Female	3.09 (1.15–8.28)	0.025	2.89 (1.05–7.92)	0.038
**Age**	18–30 years	1			
	>30 years	1.73 (0.64–4.64)	0.272	1.62 (0.59–4.45)	0.344
**Educ level**	0–7 years	1			
	>7 years	1.26 (0.44–3.59)	0.658	1.40 (0.48–4.02)	0.540
**Type of psychosis** *	Mania	1.38 (0.54–3.51)	0.492	1.29 (0.50–3.33)	0.590
	Depression	2.40 (0.31–18.33)	0.399	1.19 (0.24–15.0)	0.532
	Schizophrenia	0.73 (0.25–2.07)	0.557	0.90 (0.30–2.65)	0.853
	Psy NOS	0.61 (0.07–4.85)	0.639	0.49 (0.05–4.81)	0.543

* Only gender, age and education level entered into the regression model for the different types of the psychosis.

**Table 5:** Cognitive function in HIV positive and HIV negative patients at 3 and 6 months of follow up.

	Baseline		3 months		6 months	
	HIV−	HIV +	HIV−	HIV+	HIV−	HIV+
	n = 322	n = 156	n = 262	n = 116	n = 205	n = 97
Normal	16 (4.97)	3 (1.92)	25 (64.10)	14 (35.90)	33(68.75)	15(31.25)
Mild	147(45.65)	52 (33.33)	130 72.22)	50 (27.78)	101(74.81)	34 (25.19)
Severe	159 49.48)	101(64.74)	107(67.30)	52 (32.70)	71 (59.66)	48 (40.34)

## Discussion

Cognitive impairment was found to be worse among HIV positive individuals with psychosis in comparison to HIV negative individuals with psychosis. The impairment was worse among females.

To our knowledge this is the first study to use a standardized neuropsychological battery of tests to compare cognitive function in HIV positive and HIV negative patients with psychosis. Most of the studies have used only the MMSE to assess the level of cognitive function, however this test does not specify the cognitive domains that may be affected [22]. Even though previous studies have shown that individuals with primary psychosis can have cognitive impairment [23], we found that the severity of the impairment is worse in HIV positive individuals even after the symptoms of psychiatric illness decrease during follow up.

As has been observed in other studies in our setting [7], [24] mania, was the commonest presentation of psychosis. Among individuals with depression, the statistical difference observed in the HIV positive males and HIV negative males could be explained by the very low numbers of individual with the disorder. However this significance was not observed when comparing the HIV positive and HIV negative groups without stratification for gender.

Males with a diagnosis of schizophrenia were more among HIV negative individuals. It has been shown that the prevalence of schizophrenia is more and also occurs earlier among males compared to females [25]. Psychosis NOS occurred more for the HIV positive population and the majority of the individuals who presented with the disorder had no prior episode of mental illness signifying a difference in the manifestation of psychosis for the HIV positive individual.

Cognitive impairment for both psychotic and non psychotic individuals occurs more often among HIV positive older patients and more so in individuals of female gender [24], [26] similar to what we observed.

Our study had a higher representation of females compared to males in the HIV positive group by almost three thirds, reflecting what is seen in most African HIV clinic settings. Females are eager in seeking care and maintaining follow up compared to their male counter parts [27], in addition they are more affected by the HIV scourge and hence tend to be more afflicted by the complications that arise from the infection [24]. However it remains important to look into other factors that may predispose females to the development of HIV associated psychosis and co- occurring cognitive impairment. For instance, theories on the neurotoxin production, specifically kynurenic acid that has been found to be higher in HIV individuals with psychosis [28], [29], have not highlighted any differences between males and females.

A number of earlier studies emphasised that cognitive impairment and psychosis were late manifestations of HIV disease [6], [30]. The onset of psychosis primarily resulting from the HIV virus attack of the brain tissue or through opportunistic infections [31], [32], [33], [34]. Recent studies including the findings of this study show that the two conditions can sometimes occur early as evidenced by the moderate level of CD4 count and the intermediate WHO stages of disease manifestation [35]. Furthermore the cognitive impairment persists even when the symptoms of psychosis improved. This finding underscores the importance of early initiation of antiretroviral therapy for HIV positive individual who develop cognitive impairment or psychosis or both conditions since there is evidence that the situation can be alleviated by this therapy [9], [36], [37].

There were some limitations to this study. Conduction of the neuropsychological assessments is usually elaborate and sometimes the participants experience interview fatigue more so if they have been started on antipsychotic treatments. However the evaluations were carried out when the patients were usually calm enough and on lower medication dosage, indeed in some situations if a participant expressed a desire to rest, the interview would be postponed to a time when they would feel comfortable to complete the evaluation. The improvement observed in performance could be a result of practice effects however the HIV positive group still performed worse than the HIV negative group. The selection of the study participants was cumulative and when the HIV negative group reached saturation, we continued with the recruitment of the HIV positive group till the total sample size was achieved. This may have affected the randomness of the sample selected. However this occurred only in the last two months of the study. We also did not test for motor performance since the patients were on antipsychotic medication whose side effects would have introduced bias in the observations made. We had an advantage of having a large sample size which could cater for some of these individual differences. There was a considerable loss to follow up by 6 months but the large sample size allowed for statistical significance to be inferred from the number that came back for re evaluation.

In summary this study compared cognitive function in HIV positive and HIV negative individuals in a cohort of individuals with psychosis. The cognitive impairment was more pronounced among the HIV positive individuals and especially so for the females. Whereas there are explanations for the higher impairment in the HIV positive group there is a need for future research to focus on the mechanism that brings about this difference with gender. Strategies that include measures for the early detection of HIV in patients with psychosis, use of non sedating antipsychotics or the early initiation of ART treatment should be in place for improved mental health care.
